# Intentional Self-Inflicted Transcranial Screwdriver Injury: A Case Report

**DOI:** 10.7759/cureus.110094

**Published:** 2026-06-02

**Authors:** Matthew T Montgomery, Caleb W Brown, Jennifer M Osher, Bracken Burns, Lou Smith

**Affiliations:** 1 Department of Surgery, East Tennessee State University Quillen College of Medicine, Johnson City, USA; 2 Department of Surgery, University of Tennessee Medical Center, Knoxville, USA

**Keywords:** artifact-limited ct, intracranial screwdriver injury, low-velocity penetrating brain injury, self-inflicted cranial trauma, transcranial foreign body

## Abstract

Transcranial screwdriver injuries are rare forms of low-velocity penetrating cranial trauma that are most often described after interpersonal violence and may result in neurologic impairment or death. These injuries can be difficult to recognize when external wounds are small and may be incompletely characterized on initial imaging, particularly when retained metal produces artifact. Plain radiography and computed tomography (CT) are useful for defining foreign-body trajectory and guiding operative planning, but the full severity of injury may only become apparent through serial imaging and surgical exploration.

A 61-year-old male patient with schizophrenia, methamphetamine use, and prior self-inflicted injuries sustained a self-inflicted penetrating right-sided cranial injury after using a rock to drive a screwdriver into his skull. He presented with the screwdriver in place and dense left hemiplegia. Initial skull radiography demonstrated a retained transcalvarial metallic foreign body and an additional metallic density raising concern for a detached screwdriver tip. Admission noncontrast CT clarified that the second density represented a retained bullet fragment from prior injury and demonstrated a penetrating right frontoparietal foreign body with small foci of subarachnoid hemorrhage and marked streak artifact. Emergent right craniotomy with foreign-body removal revealed additional epidural and subdural hematomas not fully appreciated on initial CT and confirmed a tract extending through the right frontal motor region, across the falx, and into the left parietal region. Serial postoperative CT and MRI demonstrated the expected evolution of the injury without a discrete abscess. His hemiplegia improved during a prolonged hospital stay, with left-sided strength improving to 3/5 at discharge.

Although screwdriver-related cranial penetration has been described previously, this case is notable for its intentional self-inflicted mechanism, artifact-limited imaging, operative clarification of injury severity, multidisciplinary management, and neurologic recovery despite severe initial deficits. This case highlights the value of staged imaging, controlled operative exploration, and postoperative surveillance in low-velocity penetrating cranial trauma, particularly when retained metallic artifact limits complete radiographic characterization.

## Introduction

Nonmissile penetrating brain injury is uncommon outside of military operations. These injuries may involve the orbit, skull base foramina, or vulnerable calvarial regions and can result in vascular injury, cerebrospinal fluid leak, infection, hemorrhage, or delayed neurologic deterioration [1,2]. Civilian low-velocity penetrating brain injury is heterogeneous and may occur after interpersonal violence, accidental trauma, or self-inflicted injury. These injuries may be missed on initial examination when the external wound is small or the clinical history is limited. Civilian low-velocity cranial penetration has been reported with knives, nails, screwdrivers, and other improvised sharp objects. Reported trajectories often involve anatomically vulnerable regions such as the orbit, skull base, temporal region, and thin calvarial segments, where relatively low-velocity objects may still violate the cranial vault and injure vascular or eloquent cortical structures; therefore, optimal management relies on careful imaging assessment, operative planning, and follow-up surveillance for secondary complications [1-3]. Radiographic characterization may be significantly affected when a retained metallic foreign body is present. Specifically, screwdriver-related transcranial injury is particularly rare, with limited reported cases [4-8]. These injuries are often severe and potentially fatal because of damage to critical intracranial anatomy [8]. We present an intentional, self-inflicted transcranial screwdriver injury in which initial radiographic assessment incompletely characterized the full extent of intracranial injury because of retained metallic artifact. This case emphasizes the role of staged imaging, controlled operative exploration, and postoperative surveillance in defining injury severity and monitoring for delayed infectious and vascular complications.

## Case presentation

A 61-year-old man with a known history of schizophrenia, suicidal ideation, methamphetamine use, and prior self-inflicted injuries, including upper-extremity lacerations and a prior cranial gunshot injury with retained bullet fragment, presented to a level I trauma center with a screwdriver embedded in the right side of his head. Chart review documented multiple recent emergency visits for altered mental status, upper-extremity lacerations, and an episode of arson. Prehospital history indicated that the patient used a rock to drive the screwdriver into his skull, although the exact circumstances were limited by available history. The patient stated that he was attempting to silence auditory hallucinations.

**Figure 1:**
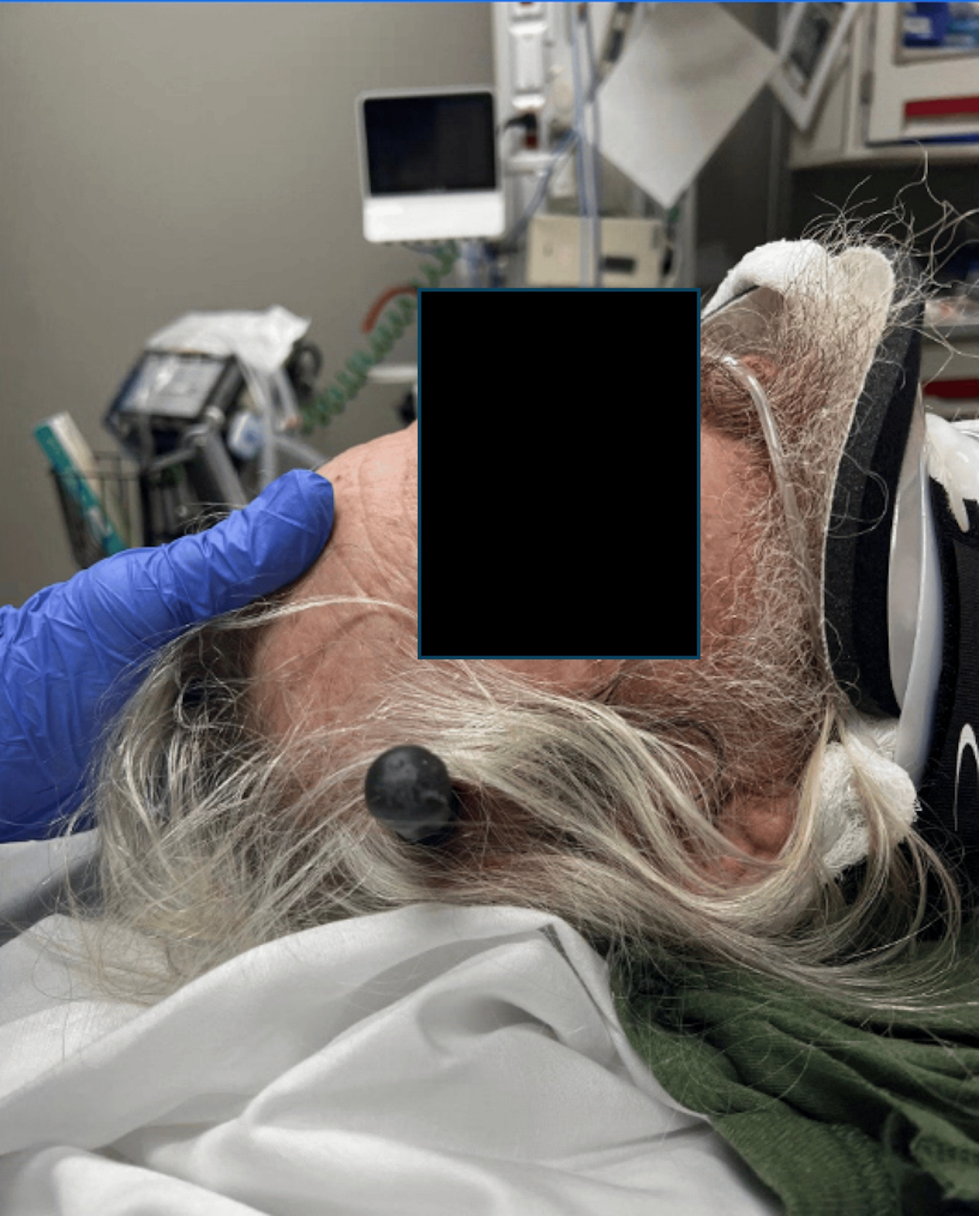
Clinical photograph at presentation demonstrating the retained screwdriver in the right temporoparietal region

On arrival, the patient was hemodynamically stable with a reported Glasgow Coma Scale (GCS) score of 13, with spontaneous eye opening, inappropriate words, and the ability to follow motor commands on the right side only. Pupils were equal, round, and reactive. Airway was patent, breath sounds were normal bilaterally, circulation was intact, and there was no active external hemorrhage. Physical examination demonstrated the screwdriver penetrating the right temporoparietal skull to the handle, with no exposed metal externally (Figure 1). Neurologically, he moved the right side of his body, had no observed movement of the left upper extremity, and demonstrated only minimal movement of the left lower extremity. Formal motor grading was limited by participation and mental status. Sensory examination was limited by participation. There were healing sutured lacerations of the upper extremity from a recent self-inflicted injury. No seizure activity was reported. Tetanus vaccination and levetiracetam 1000 mg were administered in the emergency department. Levetiracetam 1000 mg was also administered preoperatively before incision. Levetiracetam was used for seizure prophylaxis in the setting of penetrating cranial trauma with cortical involvement, consistent with local institutional practice for patients at high risk of posttraumatic seizures.

**Figure 2:**
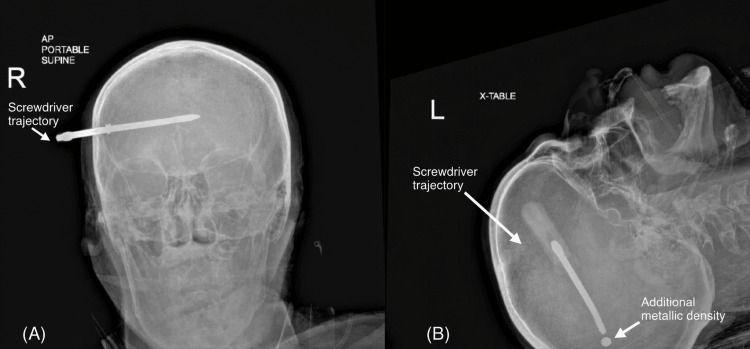
Skull radiographs demonstrating retained screwdriver and additional metallic density (A) Anteroposterior skull radiograph demonstrating the retained screwdriver traversing the cranial vault. (B) Lateral skull radiograph demonstrating the oblique depth of penetration and an additional metallic density near the distal end of the penetrating object, initially concerning for a detached screwdriver component and later clarified on CT as a retained bullet fragment from prior injury

Plain skull radiography prompted suspicion regarding the depth and extent of injury. Anteroposterior and lateral skull films demonstrated a long metallic foreign body entering the right temporoparietal calvarium and extending across the cranial vault, with an additional metallic density visible near the distal end of the penetrating object (Figures 2A-2B). This raised concern for a fractured and retained screwdriver tip. At this stage, the differential diagnosis included a retained penetrating screwdriver with a possible detached metal tip, associated intracranial hemorrhage, possible transfalcine extension, vascular injury, and other occult associated injury not yet fully characterized.

**Figure 3:**
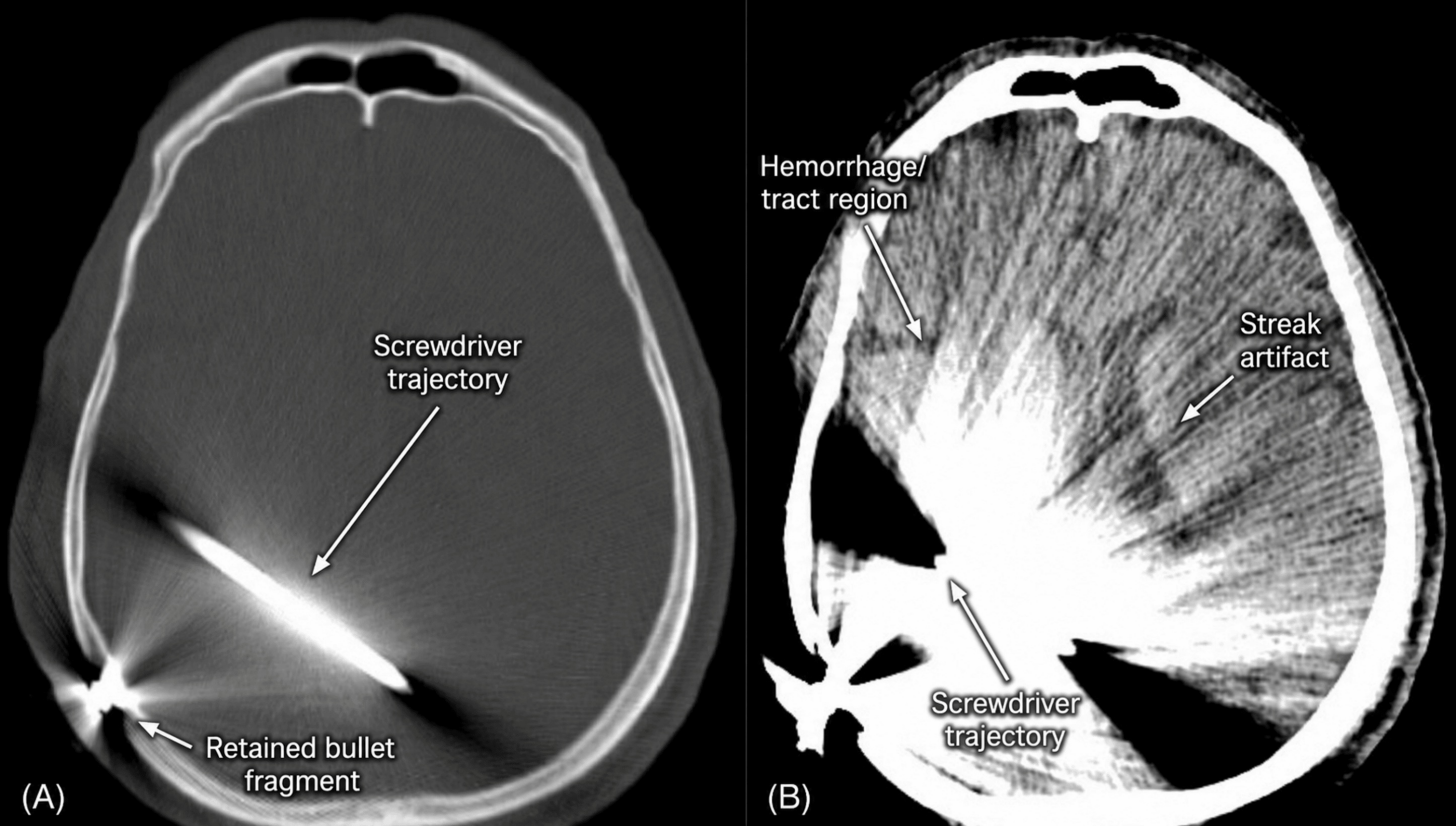
Admission CT demonstrating retained metallic foreign body and artifact-limited assessment (A) Admission axial noncontrast CT of the head on bone window demonstrating the screwdriver trajectory through the right frontoparietal calvarium and the retained bullet fragment from prior injury. (B) Admission axial noncontrast CT of the head on brain window demonstrating the screwdriver trajectory, marked streak artifact, and adjacent hemorrhage/tract region, limiting characterization of the surrounding brain parenchyma and extra-axial spaces

Admission noncontrast CT of the head demonstrated a metallic foreign body traversing the right frontoparietal calvarium into the right parietal lobe, producing small foci of predominantly right hemispheric subarachnoid hemorrhage without significant initial midline shift or mass effect (Figures 3A-3B). The metallic streak artifact significantly limited the characterization of the adjacent parenchyma and extra-axial spaces. Importantly, CT also revised the initial radiographic concern regarding a possible detached screwdriver tip: the second metallic density seen on plain films was clarified to be a retained bullet fragment from a prior injury located in the right parietal region and unrelated to the acute screwdriver penetration. Thus, admission CT excluded a broken contemporary foreign-body component while confirming intracranial penetration and associated hemorrhage.

Given the penetrating cranial injury, focal neurologic deficit, and radiographic confirmation of intracranial violation, the neurosurgical team proceeded with emergent operative exploration. Emergency consent was assumed because the patient was intubated, and no power of attorney was available. The patient was transported emergently to the operating room and placed under general endotracheal anesthesia. A Foley catheter was placed, the scalp was clipped, and the patient was placed in a Mayfield skull clamp. He was positioned in the left lateral decubitus position with the head turned to expose the right craniotomy site and the protruding screwdriver. After positioning and exposure of the right-sided operative field, the screwdriver was withdrawn and sent to pathology for gross inspection. The operative site was then prepared with alcohol and ChloraPrep and draped in a sterile fashion. Before incision, the patient received tranexamic acid 1000 mg, levetiracetam 1000 mg, and vancomycin 1250 mg.

A trauma-flap-type scalp incision was marked and injected with 1% lidocaine with 1:100,000 epinephrine. Incision was made with a #15 blade and carried through the skin, subcutaneous tissue, galea, and pericranium. The scalp and temporalis muscle were dissected from the skull using Bovie cautery and reflected anteriorly. A bullet fragment from a remote prior suicide attempt was identified embedded in the right parietal outer table and diploë, removed with a small curved curette, and sent to pathology for gross inspection. The superior sagittal suture, coronal suture, lambdoid suture, mastoid process, superior nuchal line, and inion were identified as landmarks. Six burr holes were created using a Stryker drill and perforator. The dura was dissected from the inner table using Woodson and Penfield dissectors, and a cranial flap was created using a Stryker drill with a side-cutting bit and footplate attachment.

After elevation of the cranial flap, an epidural hematoma was encountered and evacuated. A small durotomy was created with a #15 blade and extended with Adson forceps and Metzenbaum scissors to create a dural flap, exposing the right cerebral hemisphere. No active bleeding was identified. The screwdriver entry point was identified through the right frontal lobe in the motor strip. Subdural hematoma overlying the right frontal and parietal lobes was evacuated. Cotton patties were used to protect the cerebral cortex, and the superior sagittal sinus and falx were exposed within the interhemispheric fissure. Exploration identified the area where the screwdriver penetrated the falx into the left parietal lobe. No active hemorrhage or large vessel injury was identified.

The wound was irrigated with copious sterile saline solution, and hemostasis was obtained. The dura was reapproximated using 4-0 Nurolon in simple interrupted and running fashion, and a 5 × 7 DuraMatrix onlay was placed over the dura. The cranial flap was returned to its anatomic position and secured to the skull using titanium plates and screws. The scalp and temporalis muscle were returned to anatomic position. The temporalis fascia and galea were reapproximated with 0 Vicryl, and staples were applied to the skin. The patient tolerated the procedure without complication. Estimated blood loss was 200 mL. Final operative findings included removal of a bullet from the right parietal skull related to remote injury and a screwdriver tract traversing the right frontal motor strip, parietal lobe, and falx into the left parietal lobe without large vessel injury. Exact craniotomy dimensions were not available from the operative report. Intraoperative culture acquisition and use of intraoperative navigation or imaging were not documented.

The operative findings expanded the perceived severity of the injury beyond the initial CT impression. While admission CT demonstrated penetrating injury with small-volume subarachnoid hemorrhage and no significant initial mass effect, operative exploration revealed additional epidural and subdural hematomas and a transfalcine tract involving the right frontal motor strip and left parietal lobe. These findings provided a more complete anatomic explanation for the patient’s dense left-sided weakness. In this case, a retained metallic artifact limited complete radiographic assessment, and controlled operative exploration provided a more complete characterization of the intracranial injury.

Dedicated vascular imaging with CT angiography, MR angiography, or catheter angiography was not performed during hospitalization. On admission, a metallic streak artifact from the retained screwdriver limited evaluation of the surrounding region, and the patient proceeded directly to emergent operative exploration due to intracranial violation and a major focal neurologic deficit. Intraoperatively, no active hemorrhage or large vessel injury was identified. Serial postoperative CT imaging demonstrated no delayed hemorrhagic progression, and subsequent MRI described normal vascular flow voids without significant vasculopathy. Delayed vascular imaging after foreign-body removal was not documented.

The first postoperative CT, obtained on postoperative day 1, demonstrated interval right craniotomy and foreign-body removal. There was a small amount of right-sided extra-axial blood, moderately large extra-axial air, scattered petechial hemorrhage, persistent subarachnoid blood, and hemorrhage along the foreign-body tract in the right hemisphere. There was no evidence of herniation.

CT on postoperative day 3 demonstrated no substantial new interval hemorrhage and improved postoperative and posttraumatic changes. Persistent but improving mild parenchymal hematoma was present in the superior right frontal lobe. The right supratentorial subdural fluid and edema-pneumocephalus collection had decreased to 7 mm from 11 mm, the minimal subarachnoid hemorrhage had improved, and the leftward midline shift had improved from 4 mm to 2 mm according to the radiology report.

**Figure 4:**
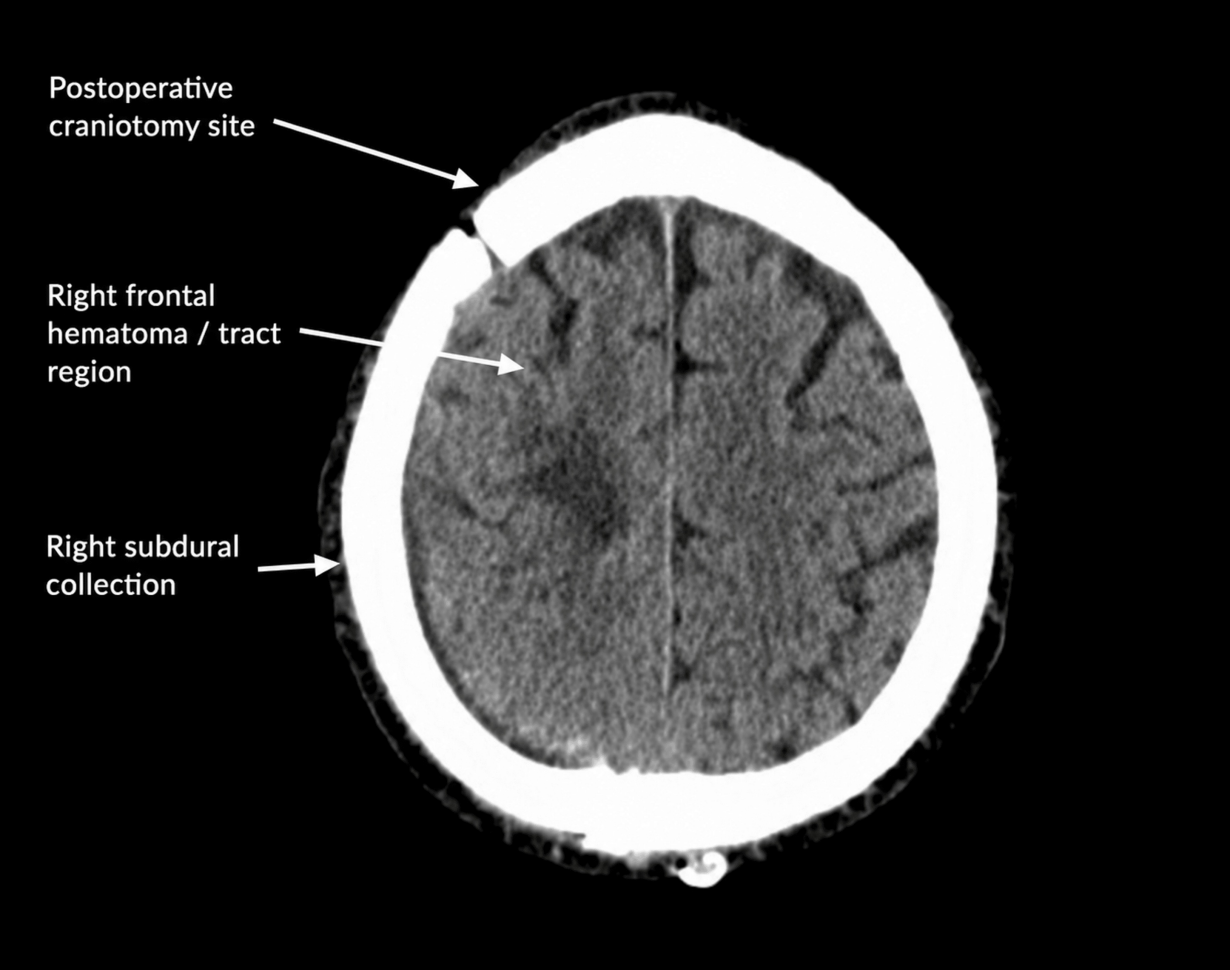
Postoperative day 25 CT demonstrating interval evolution of injury Noncontrast axial CT of the head on postoperative day 25 demonstrating postoperative craniotomy changes, expected interval evolution of the right frontal hematoma/tract region, and persistent but improving right convexity subdural collection. No new acute intracranial abnormality was identified

CT on postoperative day 10 demonstrated postoperative changes of right parietal craniotomy, expected evolution of the right frontal lobe hemorrhage, mild surrounding right frontal lobe edema, and 5 mm right-to-left midline shift without worsening intracranial hemorrhage. CT on postoperative day 25 demonstrated no significant acute intracerebral abnormality, a persistent but improving right supratentorial mixed-density subdural collection with mild pneumocephalus measuring 0.5 cm, continued expected evolution of the late subacute right superior frontal lobe hematoma, and an improving leftward midline shift from 5 mm to 3 mm (Figure 4). 

In total, five noncontrast CT head studies were obtained during hospitalization, including the admission CT on June 30, 2025, and four postoperative CT scans on postoperative days 1, 3, 10, and 25. These studies demonstrated expected postoperative and posttraumatic evolution without significant delayed hemorrhagic progression and, when interpreted alongside the patient’s clinical improvement, did not alter clinical management.

Infectious disease consultation guided antimicrobial management because of the high risk for cerebral abscess in the setting of contaminated penetrating cranial trauma with retained foreign material and cortical violation. Initial laboratory workup showed a leukocyte count of 7.8 × 10³/µL and creatinine of 0.8 mg/dL. The patient initially received ceftriaxone 2 g IV every 12 hours and vancomycin 1250 mg IV every 24 hours beginning June 30, 2025. Meropenem 2 g IV every eight hours was started on July 4, 2025. Vancomycin dosing was later adjusted to 1 g IV every 12 hours on July 14, 2025. On July 25, 2025, IV meropenem and IV vancomycin were discontinued, and oral levofloxacin 750 mg daily was started to complete therapy through July 30, 2025. On July 29, 2025, the infectious disease was reconsulted for a persistent pruritic maculopapular rash despite discontinuation of IV vancomycin and meropenem. Levofloxacin was discontinued, and oral prednisone 20 mg daily for three to five days, with diphenhydramine 25 mg every six hours as needed for itching, was recommended.

MRI brain without contrast on August 9, 2025, demonstrated right craniotomy changes with associated extra-axial fluid likely reflecting postoperative epidural blood and a small amount of blood products in the posterior right frontal lobe consistent with the history of penetrating injury. No acute finding or significant interval change was identified, and normal vascular flow voids were described. MRI brain without contrast on August 13, 2025, demonstrated continued resolution of the right frontoparietal junction hemorrhage and a stable right-sided subdural fluid collection without mass effect. Contrast-enhanced MRI on the same date demonstrated slight enhancement surrounding the resolving hematoma, favored to be reactive in nature, with no discrete abscess. Right-sided subdural fluid collection with dural enhancement was also noted and favored related to prior trauma and surgery; subdural empyema could not be excluded, but was considered less likely. CT head with and without contrast on August 30, 2025, showed no new or worsening intracranial abnormality, stable small-volume right convexity subdural fluid collection, and no new or worsening enhancing mass lesion. A follow-up MRI of the brain with and without contrast in four to six weeks was recommended.

Clinically, the patient remained lethargic on postoperative day 1 with persistent left hemiplegia. By postoperative day 3, he was intermittently following commands and answering questions appropriately. Over a 78-day hospitalization, he demonstrated gradual neurologic improvement in cognition, speech, and mobility. His left hemiplegia persisted, but left-sided active mobility improved slowly with the use of a left upper extremity hand contracture prevention splint and a left lower extremity Podus boot. At discharge, he was alert and oriented with a GCS of 15, right-sided strength of 5/5, and left-sided strength of 3/5. He could ambulate approximately 50 feet with a front-wheeled walker and assistance, but continued to demonstrate poor control of the left lower extremity, decreased left-sided awareness, balance impairment, and poor safety insight. Inpatient rehabilitation remained recommended because of persistent left-sided weakness, impaired mobility, and safety concerns, but placement was unsuccessful, and the patient refused continued inpatient rehabilitation efforts. He reported that he planned to live in a building on his property with running water and that friends would assist him. Durable medical equipment and prescriptions were ordered. 

Psychiatry followed the patient during hospitalization because of schizophrenia, suicidal ideation, delusional beliefs, hallucinations, and the psychotic context of the self-inflicted injury. He was initially on suicide precautions with a recommendation for inpatient psychiatric treatment. Earlier psychiatric assessments documented ongoing suicidal ideation and delusional beliefs that he needed to die to protect his family. His psychiatric medication regimen was adjusted during hospitalization because of persistent delusional symptoms and concern for poor medication compliance, with future consideration of long-acting injectable antipsychotic therapy. His psychiatric course improved with medical management, and the sitter was discontinued on August 22, 2025. On the day of discharge, psychiatry documented that he was alert and oriented to person, place, time, and situation, calm and cooperative, and maintained good eye contact. He reported anxiety related to the prolonged admission and difficulty sleeping because of confinement to his room, but denied suicidal ideation, homicidal ideation, and hallucinations. On discharge examination, mood, behavior, thought content, and judgment were documented as normal, and thought content did not include suicidal or homicidal ideation or plan. Psychiatric consult services determined that further inpatient psychiatric hospitalization was not indicated at discharge, and outpatient psychiatry follow-up was arranged.

Staples were removed by neurosurgery on August 1, 2025. Neurosurgery documented on August 14, 2025, that the MRI brain was stable, there was no neurosurgical intervention indicated, and the patient was acceptable for discharge from a neurosurgical standpoint with continued levetiracetam, twice-daily Hibiclens incision care, incision open to air, and neurosurgery follow-up in two weeks. At discharge, levetiracetam 500 mg twice daily was prescribed through December 15, 2025, for seizure prophylaxis after penetrating cortical injury. Neurosurgery later recommended repeat MRI brain with and without contrast in four to six weeks, followed by clinic follow-up. Outpatient follow-up with psychiatry and neurosurgery was arranged. Outpatient infectious disease follow-up and outpatient rehabilitation follow-up were not documented in the available discharge materials. Subsequent documentation states that the patient declined follow-up MRI and neurosurgery follow-up, and later canceled radiology and neurosurgery visits.

A chronological summary of the patient’s clinical course, imaging, antimicrobial therapy, neurologic recovery, psychiatric reassessment, and discharge disposition is provided in Table 1.

**Table 1:** Clinical, imaging, antimicrobial, neurologic, and psychiatric timeline CT: computed tomography; MRI: magnetic resonance imaging; POD: postoperative day; GCS: Glasgow Coma Scale; IV: intravenous

Time point	Event
June 30, 2025, admission	Patient presented as a level I trauma with a screwdriver embedded in the right temporoparietal skull, GCS 13, and dense left-sided weakness.
June 30, 2025, skull radiography	Anteroposterior and lateral skull radiographs demonstrated a retained metallic foreign body traversing the cranial vault and an additional metallic density initially concerning for a detached screwdriver component.
June 30, 2025, admission CT	Non-contrast CT head demonstrated right frontoparietal cranial penetration into the right parietal lobe, small foci of predominantly right hemispheric subarachnoid hemorrhage, no significant initial midline shift or mass effect, and metallic streak artifact limiting evaluation. CT clarified that the second metallic density was a retained bullet fragment from prior injury.
June 30, 2025, emergency department	Tetanus vaccination and levetiracetam 1000 mg were administered. The patient was intubated for airway protection and preparation for emergent operative intervention.
June 30, 2025, surgery	Patient underwent emergent right craniotomy for evacuation of epidural and subdural hematomas and removal of the retained screwdriver. A prior retained bullet fragment was also removed from the right parietal skull. Operative findings demonstrated a screwdriver tract through the right frontal motor strip, parietal lobe, falx, and into the left parietal lobe without large vessel injury.
June 30, 2025, antimicrobial therapy	Ceftriaxone 2 g IV every 12 hours and vancomycin 1250 mg IV every 24 hours were started.
July 1, 2025, POD1 CT	CT head demonstrated interval right craniotomy and foreign-body removal, small right-sided extra-axial blood, moderately large extra-axial air, scattered petechial hemorrhage, persistent subarachnoid blood, and hemorrhage along the foreign-body tract without herniation.
July 3, 2025, POD3 CT	CT head demonstrated no substantial new interval hemorrhage and improving postoperative/post-traumatic changes. Right supratentorial subdural fluid and edema-pneumocephalus collection decreased from 11 mm to 7 mm, minimal subarachnoid hemorrhage improved, and leftward midline shift improved from 4 mm to 2 mm.
July 4, 2025	Meropenem 2 g IV every 8 hours was started.
July 10, 2025, POD10 CT	CT head demonstrated expected evolution of the right frontal lobe hemorrhage, mild surrounding edema, and 5 mm right-to-left midline shift without worsening intracranial hemorrhage.
July 14, 2025	Vancomycin dosing was adjusted to 1 g IV every 12 hours.
July 25, 2025, POD25 CT	CT head demonstrated no significant acute intracerebral abnormality, persistent but improving right supratentorial mixed-density subdural collection with mild pneumocephalus measuring 0.5 cm, continued expected evolution of the late subacute right superior frontal lobe hematoma, and improved leftward midline shift from 5 mm to 3 mm.
July 25, 2025	IV meropenem and IV vancomycin were discontinued. Oral levofloxacin 750 mg daily was started to complete therapy through July 30, 2025.
July 29, 2025	Infectious disease was reconsulted for persistent pruritic maculopapular rash despite discontinuation of IV vancomycin and meropenem. Levofloxacin was discontinued, and oral prednisone 20 mg daily for three to five days with diphenhydramine 25 mg every six hours as needed for itching was recommended.
August 1, 2025	Staples were removed by neurosurgery.
August 9, 2025, MRI without contrast	MRI brain demonstrated right craniotomy changes with associated extra-axial fluid likely reflecting postoperative epidural blood and a small amount of blood products in the posterior right frontal lobe, without acute finding or significant interval change. Normal vascular flow voids were described.
August 13, 2025, MRI without contrast	MRI brain demonstrated continued resolution of the right frontoparietal junction hemorrhage and stable right-sided subdural fluid collection without mass effect.
August 13, 2025, MRI with contrast	Contrast-enhanced MRI demonstrated slight enhancement surrounding the resolving hematoma, favored reactive, without discrete abscess. Right-sided subdural fluid collection with dural enhancement was favored related to prior trauma and surgery; subdural empyema could not be excluded but was considered less likely.
August 14, 2025	Neurosurgery documented stable MRI brain, no neurosurgical intervention indicated, continued levetiracetam, twice-daily Hibiclens incision care, incision open to air, and neurosurgery follow-up in two weeks.
August 22, 2025	Sitter was discontinued after psychiatric improvement.
August 30, 2025, CT with and without contrast	CT head demonstrated no new or worsening intracranial abnormality, stable small-volume right convexity subdural fluid collection, and no new or worsening enhancing mass lesion. Follow-up MRI brain with and without contrast in four to six weeks was recommended.
September 16, 2025, discharge	Patient was alert and oriented with GCS 15 and persistent functional impairment. Inpatient rehabilitation remained recommended but was refused after unsuccessful placement efforts. Durable medical equipment and prescriptions were ordered.
September 16, 2025, discharge medications/follow-up	Levetiracetam 500 mg twice daily was prescribed through December 15, 2025, for seizure prophylaxis after penetrating cortical injury. Outpatient psychiatry and neurosurgery follow-up were arranged.
After discharge	Subsequent documentation stated that the patient declined follow-up MRI and neurosurgery follow-up and later canceled radiology and neurosurgery visits.

## Discussion

This case adds to the small body of literature describing screwdriver-related cranial penetration and emphasizes how the apparent severity of injury may evolve as imaging and operative findings accumulate. Smrkolj et al. described two screwdriver intracranial injuries, both fatal because of arterial injury with subsequent brain ischemia and edema [4]. Tutton et al. reported four intracranial penetrating screwdriver injuries, two fatal and two resulting in functional deficits. They observed that in half of their cases, penetration was not initially suspected because the entry wound was small and the history was limited [5]. De Tommasi et al. emphasized the value of CT, including three-dimensional reconstruction, for defining screwdriver trajectory and guiding emergency surgery in severe skull-base injury [6]. Shi et al. described a screwdriver injury passing through the right zygomatic bone to the posterior cranial fossa, in which CT-defined trajectory informed foreign-body removal and hematoma evacuation [7]. Pavlidis et al. concluded that screwdriver assaults to the cranium are uncommon and potentially severe, and that the extent of trauma depends on the dimensions of the screwdriver and the involved anatomic region [8]. Bodwal et al. also emphasized the rarity of screwdriver penetration and the role of imaging in defining the path of injury [9]. A summary of previously reported screwdriver-related cranial injury publications compared with the present case is provided in Table 2.

**Table 2:** Summary of previously reported screwdriver-related cranial injury publications and the present case CT: computed tomography; MRI: magnetic resonance imaging. This table summarizes available data from the cited publications. “Not reported” indicates that the detail was not available from the accessible article text or abstract reviewed for this manuscript

Author/year	Mechanism	Self-inflicted vs assault/other	Entry site	Trajectory	Imaging modality	Operative management	Vascular injury	Infectious complication	Neurologic outcome	Survival status
Smrkolj et al., 1995	Screwdriver-related intracranial injury	Not reported	Not reported	Intracranial penetration	Not reported	Not reported	Arterial injury reported	Not reported	Brain ischemia and edema	Fatal
Tutton et al., 2000	Intracranial penetrating screwdriver injuries	Assault	Small cranial entry wounds; specific sites varied	Intracranial penetration	Clinical evaluation and imaging; penetration initially unsuspected in some cases	Not reported	Not reported	Not reported	Functional deficits in two survivors	Two fatal; two survived with deficits
De Tommasi et al., 2006	Fall onto screwdriver	Accidental	Skull base	Severe skull-base penetration	CT with three-dimensional reconstruction	Emergency surgical removal	Not reported	Not reported	Survived; neurologic outcome not fully reported	Survived
Shi et al., 2017	Screwdriver-induced penetrating brain injury	Assault	Right craniofacial/zygomatic region	Penetration toward posterior cranial fossa	CT-defined trajectory	Foreign-body removal and hematoma evacuation	Not reported	Not reported	Successfully managed	Survived
Pavlidis et al., 2016	Screwdriver stab injuries	Mainly interpersonal violence	Variable	Craniocerebral stab wounds	Literature review	Variable	Reported across reviewed cases	Reported across reviewed cases	Variable	Mortality reported in reviewed cases
Bodwal et al., 2013	Forceful screwdriver penetration	Assault/homicide	Cranial cavity; orbital/temporal vulnerability discussed	Intracranial penetration	Plain radiography and CT with weapon in situ	Pre-autopsy imaging; operative management not applicable	Not reported	Not reported	Brought dead on arrival	Fatal
Present case	Screwdriver driven into skull with rock	Intentional self-inflicted	Right temporoparietal/right frontoparietal region	Right frontal motor strip, parietal lobe, falx, and left parietal lobe	Skull radiography, admission CT bone and brain windows, serial CT, MRI	Emergent right craniotomy, foreign-body removal, evacuation of epidural and subdural hematomas, dural repair, bone-flap replacement	No active hemorrhage or large vessel injury identified intraoperatively; dedicated vascular imaging not performed	No discrete abscess on follow-up MRI or CT	Improved from dense left hemiplegia to partial motor recovery with persistent functional impairment at discharge	Survived

The present case is notable because its principal teaching point extends beyond the rarity of injury and focuses on the progressive clarification of diagnosis and severity through staged radiographic and intraoperative evaluation. On plain radiography, the injury appeared to consist of a transcranial screwdriver with a second distal metallic component that could reasonably have represented a broken detachable tip. This initial interpretation mattered because a fractured intracranial tool fragment would have implied a more complex retained foreign-body burden. Admission CT then revised that impression by showing that the second density was a retained bullet from a remote prior injury, unrelated to the acute event. At the same time, CT confirmed penetrating intracranial trauma and subarachnoid hemorrhage, but artifact from the retained metal limited assessment of the surrounding parenchyma and extra-axial spaces.

In this patient, operative exploration was favored over observation or simple bedside extraction because the screwdriver violated the cranial vault, the patient had a major focal neurologic deficit, and imaging was limited by metallic artifact. Controlled craniotomy allowed foreign-body removal under direct visualization, evacuation of epidural and subdural hematomas, inspection for active hemorrhage, and dural repair. These goals would not have been achievable with observation alone or unplanned extraction.

Operative exploration further refined the true extent of injury. Surgery revealed epidural and subdural hematomas not fully appreciated on the admission CT, and confirmed a transfalcine tract through the right frontal motor region into the left parietal region. The CT also prompted removal of the retained bullet fragment, allowing the opportunity for MRI evaluation as needed. In this way, the case demonstrates that the severity of low-velocity penetrating cranial trauma may be underestimated or inaccurately characterized if early interpretation rests too heavily on initial imaging alone. Across prior reports, imaging has functioned not merely as confirmation of penetration but as the basis for operative planning and interpretation of severity [6,7,9].

The present case also reflects several known high-risk features of non-missile penetrating brain injury. Sweeney et al. noted that these injuries may result in vascular injury, pseudoaneurysm, arteriovenous fistula, vasospasm, cerebrospinal fluid leak, and infection [2]. Harrington et al. later showed that the most common secondary complications are vascular injury and infection and that systematic evaluation and follow-up are essential for diagnosis and management of these complications [1]. In the present patient, the tract involved the eloquent motor cortex and crossed the falx. Early imaging documented subarachnoid blood, and later imaging prompted evaluation for abscess or empyema. Although no major vascular injury was identified intraoperatively and no discrete abscess developed, the hospital course illustrates why serial reassessment remains necessary even after technically successful foreign-body removal [1,2].

Vascular injury remains an important consideration in penetrating brain injury, including pseudoaneurysm, arteriovenous fistula, vasospasm, and delayed hemorrhage [1,2]. This concern is particularly relevant in the present case because the injury crossed the falx, involved subarachnoid hemorrhage, and traversed eloquent cortical regions. Although no active hemorrhage or large vessel injury was identified intraoperatively, and serial postoperative imaging did not demonstrate delayed hemorrhagic progression, dedicated vascular imaging was not performed. Subsequent MRI described normal vascular flow voids without significant vasculopathy, but this does not replace CT angiography, MR angiography, or catheter angiography. Delayed vascular imaging after foreign-body removal was not documented. The absence of dedicated vascular imaging remains a major limitation of this report. In similar cases, vascular imaging should be considered after initial stabilization or foreign-body removal when trajectory, hemorrhage pattern, proximity to major vessels, or clinical course raises concern for arterial injury.

Infectious complications are also a major concern after penetrating cranial trauma, particularly when retained foreign material, contaminated mechanism, and cortical violation are present. Antibiotic prophylaxis in penetrating traumatic brain injury remains controversial, and published regimens vary across institutions and reports [10]. In this patient, infectious disease consultation documented high risk for cerebral abscess and guided broad-spectrum antimicrobial management based on individualized risk assessment. Initial workup showed a leukocyte count of 7.8 × 10³/µL and creatinine of 0.8 mg/dL. The patient received ceftriaxone, vancomycin, and meropenem during hospitalization, followed by oral levofloxacin to complete the planned course through July 30, 2025. Therapy was modified after the development of a persistent pruritic maculopapular rash. Follow-up MRI and CT demonstrated postoperative and post-traumatic changes without a discrete abscess.

In cases of low-velocity penetrating cranial trauma involving retained metallic foreign bodies, radiographic assessment may underestimate the true extent of injury because artifact can obscure adjacent parenchymal and extra-axial pathology [1,2]. This case suggests that when a significant neurologic deficit is present, and imaging remains limited by retained metal, early operative exploration should be considered rather than delayed observation alone. In the present patient, the full extent of injury was more clearly characterized after craniotomy revealed additional hematoma burden and transfalcine extension not fully appreciated on initial CT.

The patient’s dense left-sided weakness correlated with the operative finding of right frontal motor strip involvement and transfalcine extension. Postoperative imaging then documented the expected evolution of tract hemorrhage, subdural fluid, edema, pneumocephalus, and midline shift before gradual improvement.

Self-inflicted penetrating cranial injury is uncommon but has been described in patients with severe psychiatric illness and recurrent self-harm, often involving nails or other improvised objects rather than screwdrivers specifically [11]. In the present case, schizophrenia, suicidal ideation, prior self-inflicted trauma, and charted auditory hallucinations formed the clinical context. The retained bullet fragment identified on CT also demonstrated that the patient had survived a prior penetrating cranial injury, adding further context to the recurrent and severe pattern of self-harm. However, the neurosurgical significance of the current presentation remained centered on the staged radiographic and operative definition of injury severity.

This case also demonstrates that survival and meaningful neurologic recovery are possible despite a dramatic initial presentation. More recent reviews of low-velocity penetrating brain injury have suggested that outcomes may be better than older literature suggests when prompt imaging, operative management, antimicrobial prophylaxis, antiepileptic prophylaxis, and serial follow-up are used appropriately [1,3]. The current patient retained left-sided weakness but improved from dense hemiplegia and limited responsiveness to alert orientation, assisted ambulation, and partial motor recovery.

The patient’s discharge course also underscores that survival after low-velocity penetrating cranial trauma does not necessarily equate to complete functional recovery. Although he improved substantially from dense left hemiplegia, he remained functionally impaired at discharge with persistent left-sided weakness, impaired mobility, and safety concerns. Inpatient rehabilitation was recommended but ultimately declined after unsuccessful placement efforts and patient refusal. This limited the ability to provide intensive supervised rehabilitation despite ongoing deficits and increased the importance of outpatient neurosurgical, psychiatric, rehabilitation, and repeat imaging follow-up. Subsequent documentation that the patient declined or canceled recommended follow-up imaging and neurosurgical evaluation further highlights the difficulty of longitudinal care in patients with severe neuropsychiatric disease and traumatic brain injury.

Although screwdriver-related cranial penetration has been described previously, the present case is notable for its intentional self-inflicted mechanism, artifact-limited imaging, operative clarification of injury severity, prolonged multidisciplinary management, and neurologic recovery despite severe initial deficits.

## Conclusions

Self-inflicted transcranial screwdriver injury is rare and potentially devastating. In this patient, plain radiography helped define foreign-body presence, orientation, and extent, while CT refined the diagnosis but was limited by metallic artifact. Operative exploration revealed additional epidural and subdural hematomas and a transfalcine tract through eloquent motor cortex that were not fully appreciated on initial imaging. This case demonstrates that, in selected patients with retained metallic foreign bodies, focal neurologic deficit, and artifact-limited imaging, early operative exploration may provide important diagnostic and therapeutic value. Serial follow-up remains essential to monitor for delayed complications, including infection, abscess or empyema, delayed hemorrhage, vascular injury, and functional decline. Meaningful neurologic recovery may still be possible despite a dramatic initial presentation with severe deficits, although persistent impairment may require prolonged rehabilitation and reliable outpatient follow-up.

## References

[1] Harrington BM, Gretschel A, Lombard C, Lonser RR, Vlok AJ (2021). Complications, outcomes, and management strategies of non-missile penetrating head injuries. J Neurosurg.

[2] Sweeney JM, Lebovitz JJ, Eller JL, Coppens JR, Bucholz RD, Abdulrauf SI (2011). Management of nonmissile penetrating brain injuries: a description of three cases and review of the literature. Skull Base Rep.

[3] Cook R, Zima L, Khazaal J, Williams J (2024). Low-velocity penetrating brain injury: a review of the literature and illustrative case. Brain Inj.

[4] Smrkolj V, Balazic J, Princic J (1995). Intracranial injuries by a screwdriver. Forensic Sci Int.

[5] Tutton MG, Chitnavis B, Stell IM (2000). Screwdriver assaults and intracranial injuries. J Accid Emerg Med.

[6] De Tommasi A, Cascardi P, De Tommasi C, Luzzi S, Ciappetta P (2006). Emergency surgery in a severe penetrating skull base injury by a screwdriver: case report and literature review. World J Emerg Surg.

[7] Shi J, Mao Y, Cao J, Dong B (2017). Management of screwdriver-induced penetrating brain injury: a case report. BMC Surg.

[8] Pavlidis P, Karakasi MV, Birbilis TA (2016). Traumatic brain injury due to screwdriver assaults: literature review and case report. Am J Forensic Med Pathol.

[9] Bodwal J, Sreenivas M, Aggrawal A (2013). Intracranial penetrating injury by screw driver: a case report and review of literature. J Forensic Leg Med.

[10] Ganga A, Leary OP, Sastry RA, Asaad WF, Svokos KA, Oyelese AA, Mermel LA (2023). Antibiotic prophylaxis in penetrating traumatic brain injury: analysis of a single-center series and systematic review of the literature. Acta Neurochir (Wien).

[11] Schultz A, Wroblewski TH, Ononogbu-Uche FC (2025). Self-inflicted bilateral penetrating brain injury with a nail gun in an African American male: illustrative case. J Neurosurg Case Lessons.

